# Supplementary material for: Exposing the hidden influence of selection rules on phonon–phonon scattering by pressure and temperature tuning
Source: Nat Commun. 2021 Jun 9;12:3473. doi: 10.1038/s41467-021-23618-7 (PMC8190138; doi:10.1038/s41467-021-23618-7)
Supplement: Supplementary file 1 — Supplementary Information [file 41467_2021_23618_MOESM1_ESM.pdf]

Exposing the hidden influence of selection rules on phonon-phonon scattering by pressure and temperature tuning
Supplementary Materials

Navaneetha K. Ravichandran[1,*] and David Broido[2]
[1]*Department of Mechanical Engineering, Indian Institute of Science, Bangalore, Karnataka 560012, India*
[2]*Department of Physics, Boston College, Chestnut Hill, MA 02467, USA*

[*] navaneeth@iisc.ac.in

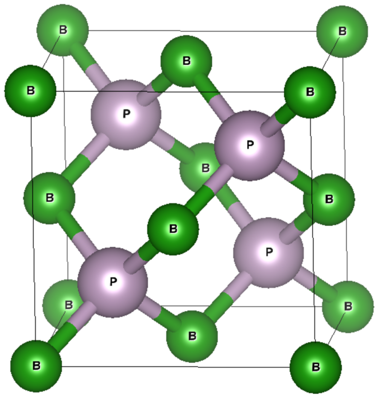

Supplementary Figure 1. **Crystal structure of the zinc blende BP.** The crystal structures of all materials and at all (T, P) conditions considered in this study (BN, BP, BAs and 3C-SiC) are zinc blende structures.

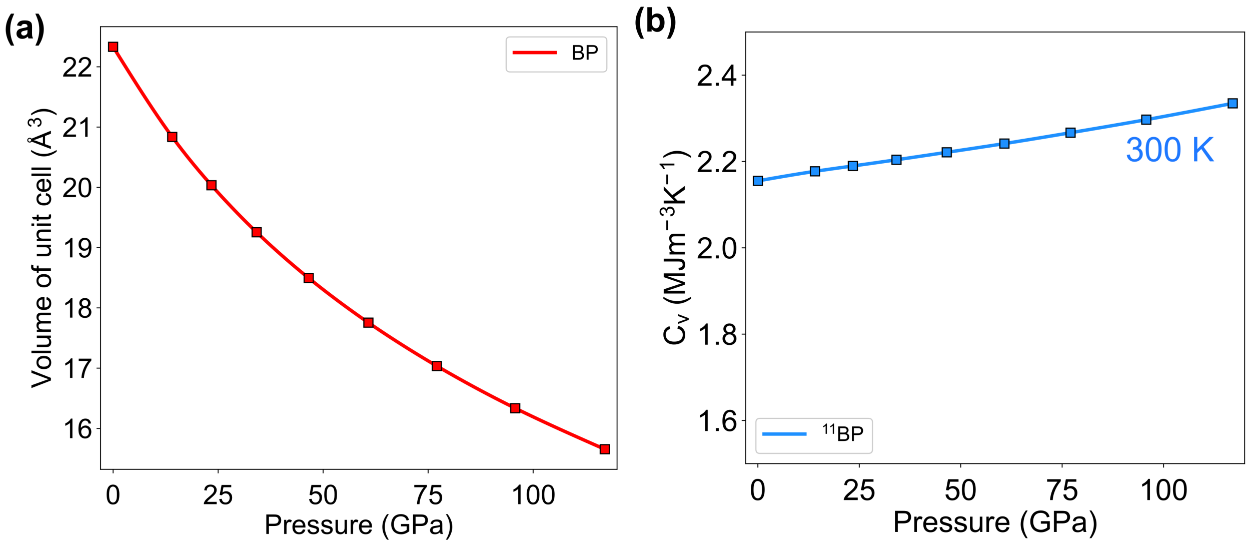

Supplementary Figure 2. **Pressure-dependent volume and volumetric heat capacity of BP. (a)** The crystal volume of BP as a function of pressure. The curves for ^{10}BP, ^{11}BP and ^{Nat}BP were almost overlapping, so only one curve for BP is shown. **(b)** The volumetric heat capacity of ^{11}BP as a function of pressure at 300 K.

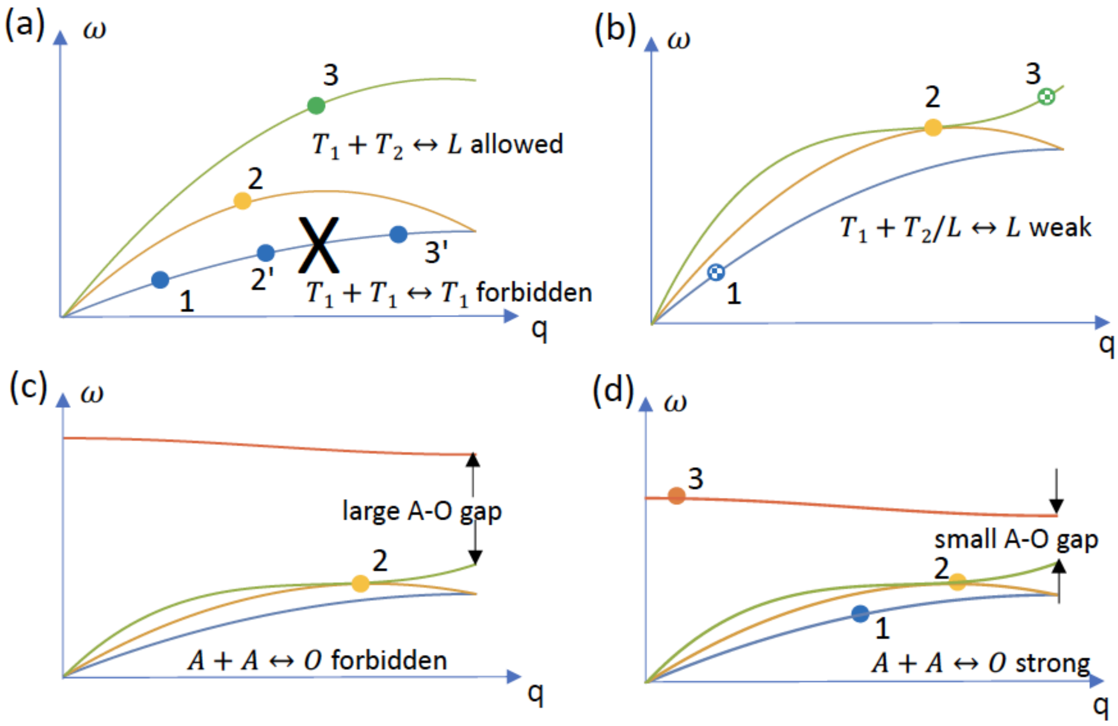

Supplementary Figure 3. **Schematic of the effects of AAA and AAO selection rules for hypothetical phonon dispersions.** **(a)** Three-phonon anharmonic decay within a single acoustic (A) phonon branch ($T_1 + T_1 \leftrightarrow T_1$) is forbidden by the AAA selection rule (marked with X). The interbranch process ($T_1 + T_2 \leftrightarrow L$) is allowed. **(b)** Hypothetical dispersions where L and T_1 branches approach each other and are degenerate at the point 2; a phonon at this point cannot decay within either L or T_2 branch, and the other AAA decay channels such as $T_1 + T_2/L \leftrightarrow L$ are weak. **(c)** If the frequency gap between the acoustic and the optic (O) phonons (A-O gap) is large, anharmonic decay involving two acoustic and one optic phonons (AAO process) is forbidden. Phonons around point 2 will have large intrinsic lifetimes, since the AAO processes are forbidden and the AAA processes are weak. **(d)** If the A-O gap is small, then the AAO processes can be strong, such as the $T_1 + T_2/L \leftrightarrow O$ process at points 1, 2 and 3 shown in the figure.

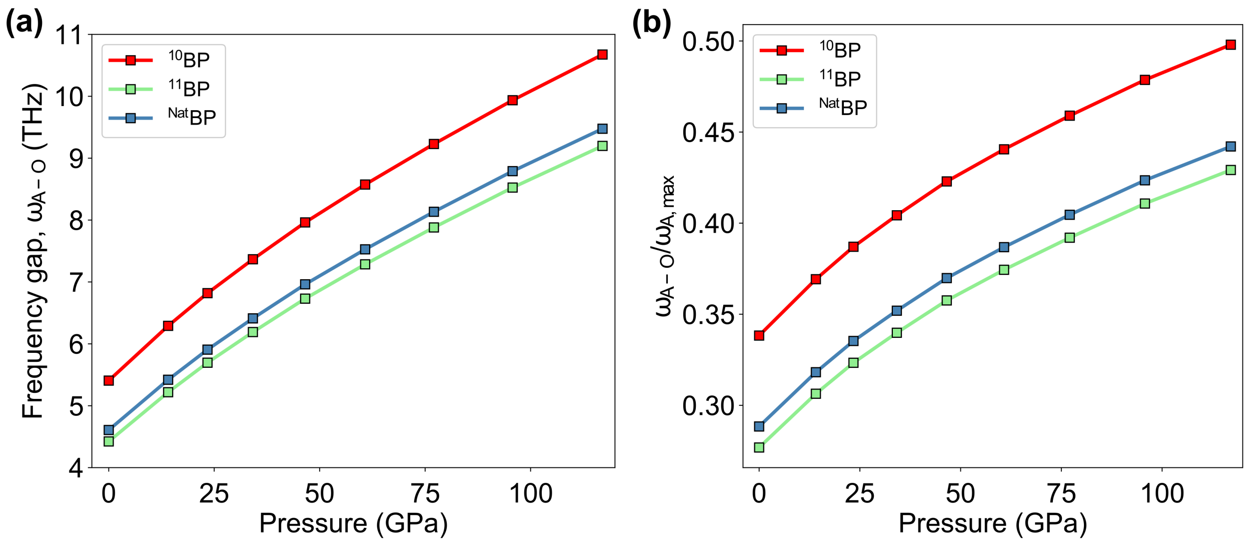

Supplementary Figure 4. **Pressure-dependent variations of the A-O gap in the dispersions of BP. (a)** The calculated A-O gap, and **(b)** the ratio of the A-O gap to the maximum acoustic phonon frequency, as a function of pressure in ^{10}BP, ^{11}BP, and ^{Nat}BP at 300 K.

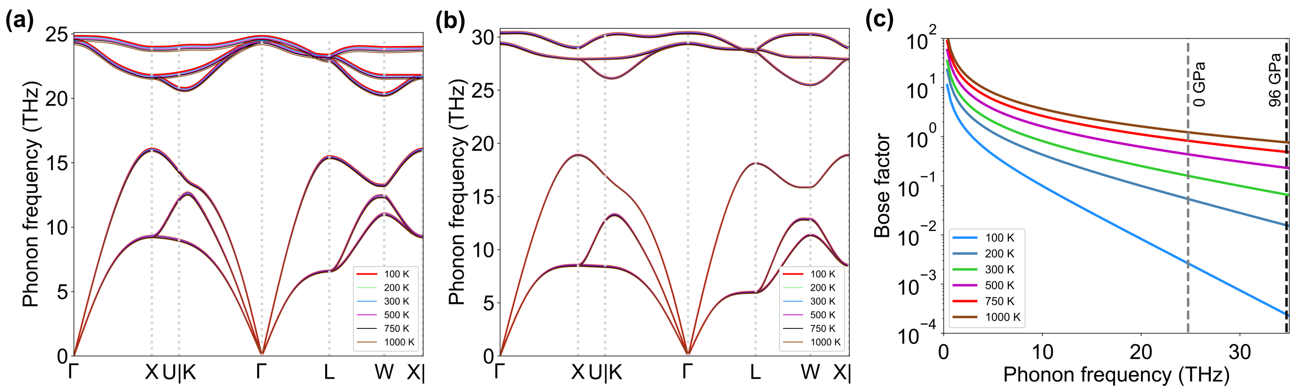

Supplementary Figure 5. **Temperature dependence of the phonon dispersions and the Bose factors of BP.** Phonon dispersions of ^{11}BP at different temperatures and at **(a)** 0 GPa and **(b)** 47 GPa. **(c)** The variation of the Bose factor with the phonon frequency at different temperatures. Dashed vertical lines indicate the maximum optic phonon frequency at the displayed pressures in ^{11}BP.

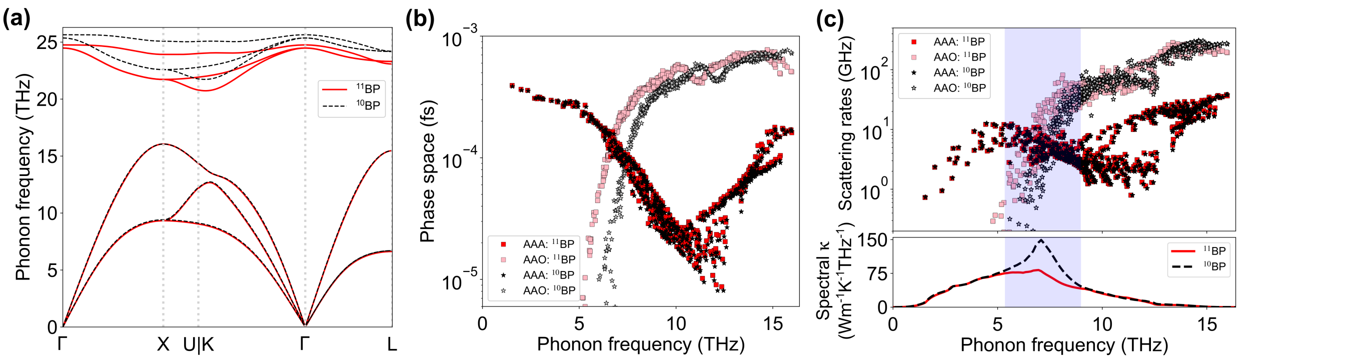

Supplementary Figure 6. **Phonon properties of ^{10}BP vs. ^{11}BP. (a)** Calculated phonon dispersions of ^{10}BP and ^{11}BP at 300 K and ambient pressure. **(b)** Phase space for AAA and AAO three-phonon scattering processes for ^{10}BP and ^{11}BP. **(c)** Scattering rates for AAA and AAO three-phonon processes (top panel) and spectral contributions to κ [$(\kappa(\nu)$] (bottom panel) for ^{10}BP and ^{11}BP. The slightly stiffer optic phonons in ^{10}BP compared to ^{11}BP shifts AAO processes to higher frequencies, thereby exposing more of the AAA phase space dip, causing weaker three-phonon scattering rates and, as a result, higher κ contributions from a narrow frequency region (shown by the blue shaded region in (c)) in ^{10}BP.

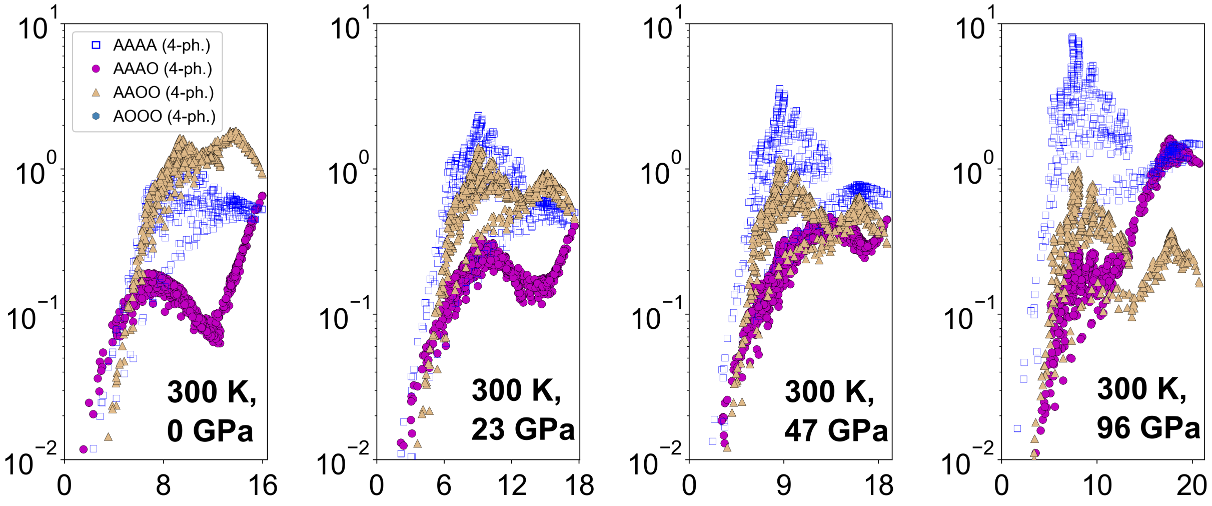

Supplementary Figure 7. **Process-wise four-phonon scattering rates for BP.** Process-wise four-phonon scattering rates for BP at T = 300 K at different pressures. AAAA scattering rates increase with increasing P and dominate at high P.

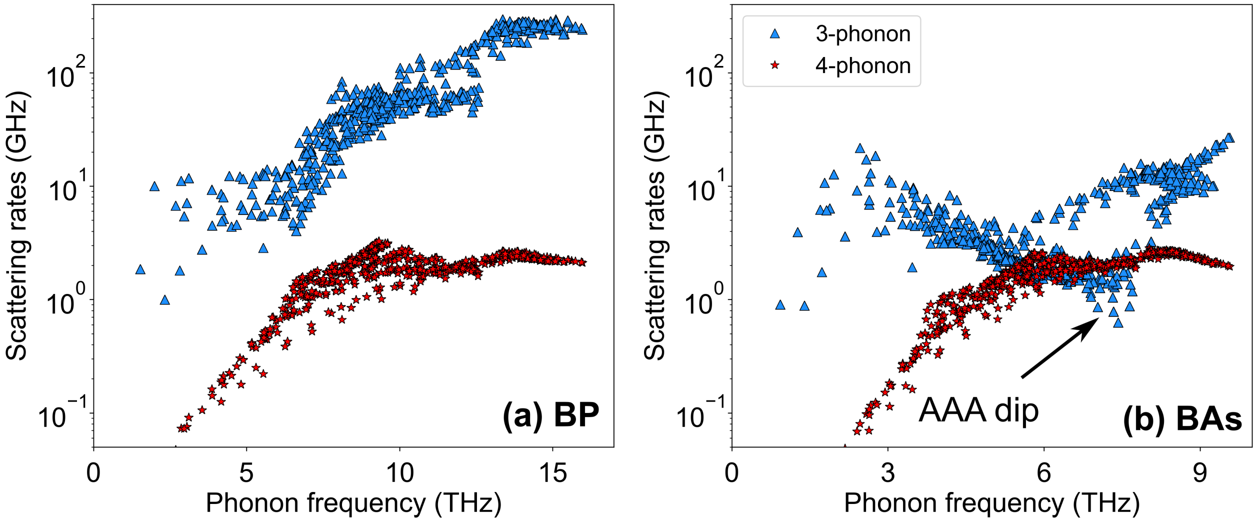

Supplementary Figure 8. **Three-phonon vs. four-phonon scattering rates of BP and BAs.** Comparison of three-phonon and four-phonon scattering rates for BP **(a)** and BAs **(b)** at 300 K and 0 GPa. The sharp dip in the three-phonon scattering rates due to the AAA selection rule is seen in BAs but hidden in BP at ambient pressure by strong AAO scattering.

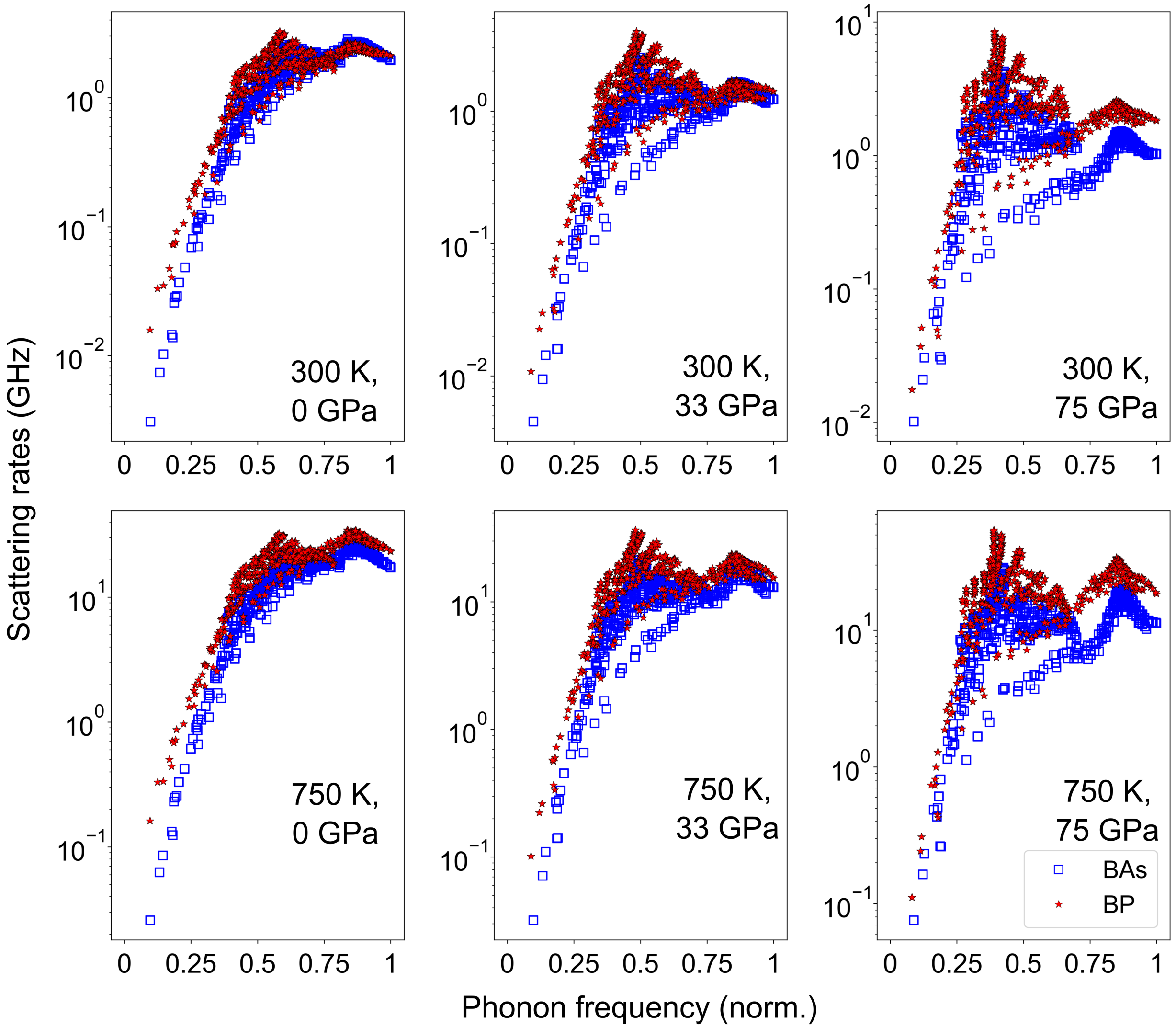

Supplementary Figure 9. **Comparison of four-phonon scattering rates for BP and BAs at different temperatures and pressures.** Overall, the four-phonon scattering rates are comparable in magnitude under all of the conditions considered in this work for BP and BAs.

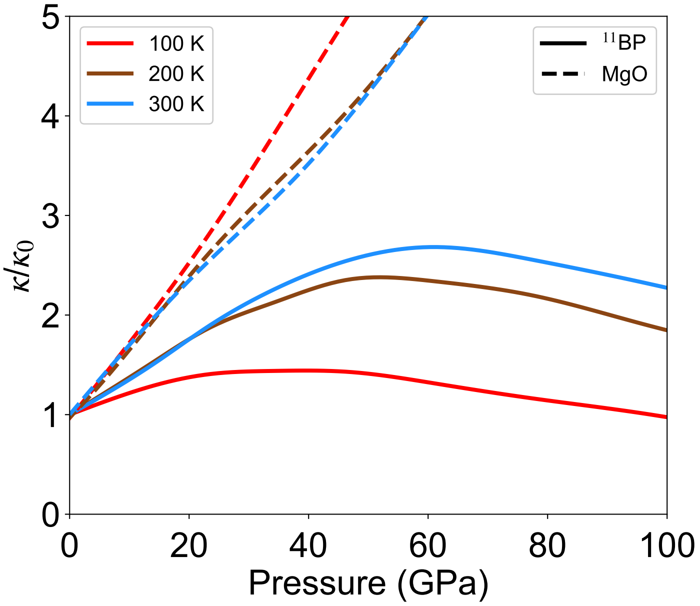

Supplementary Figure 10. **Comparison of the pressure dependencies of the scaled κ of BP and MgO at different temperatures.** Calculated $\kappa(P)$ of ^{11}BP and MgO, scaled by their zero pressure values, κ_0, at temperatures of 100 K, 200 K and 300 K. With decreasing temperatures, $\kappa(P)/\kappa_0$ decreases in ^{11}BP, in striking contrast to the behavior in MgO which shows an increase in $\kappa(P)/\kappa_0$.

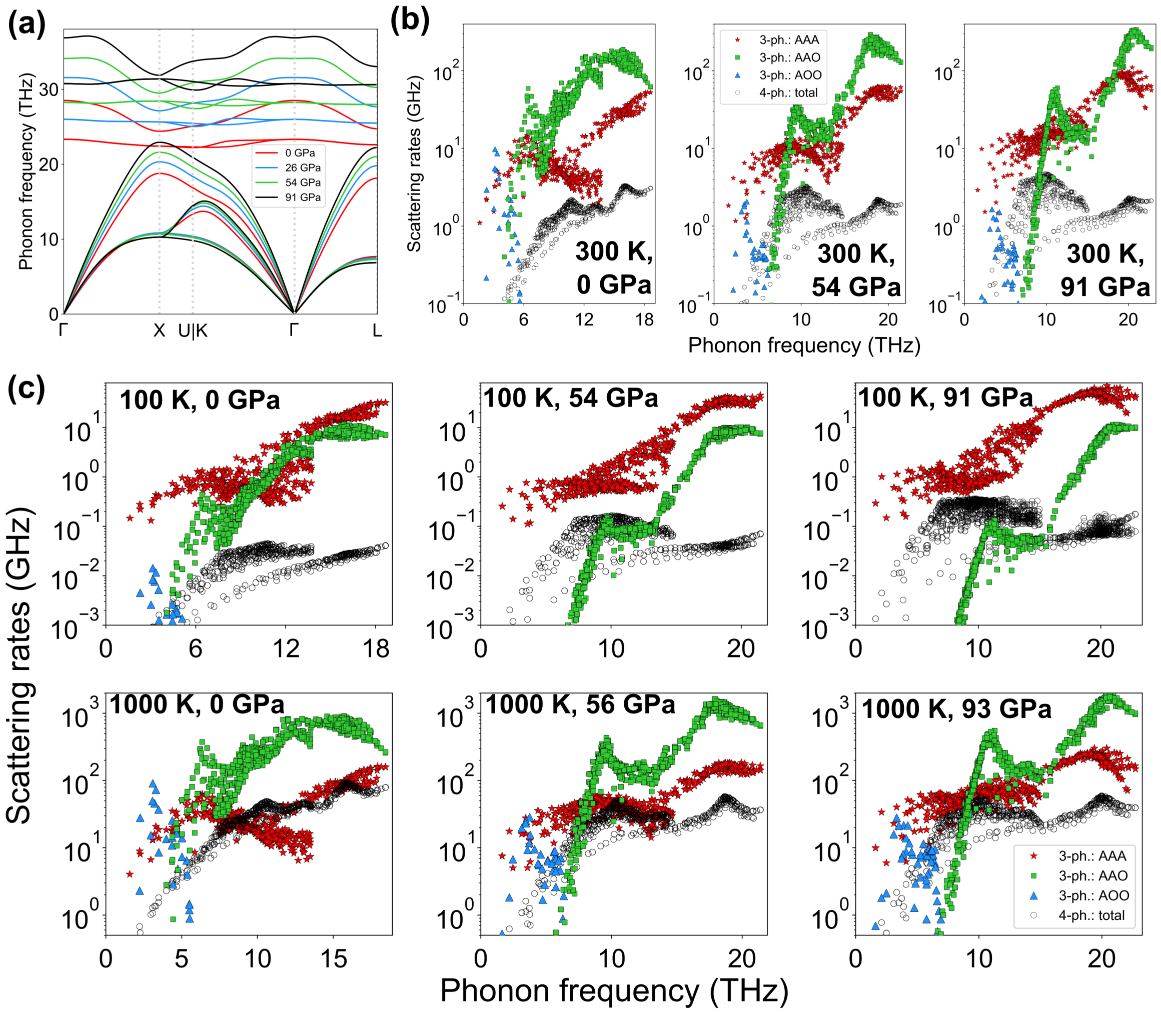

Supplementary Figure 11. **Pressure and temperature-dependent phonon dispersions and scattering rates of 3C-SiC.** **(a)** Phonon dispersions for 3C-SiC at 300 K and different pressures. **(b)** Process-wise separated three-phonon and total four-phonon scattering rates of 3C-SiC at 300 K and different pressures. **(c)** Same as (b) for 100 K and 1000 K.

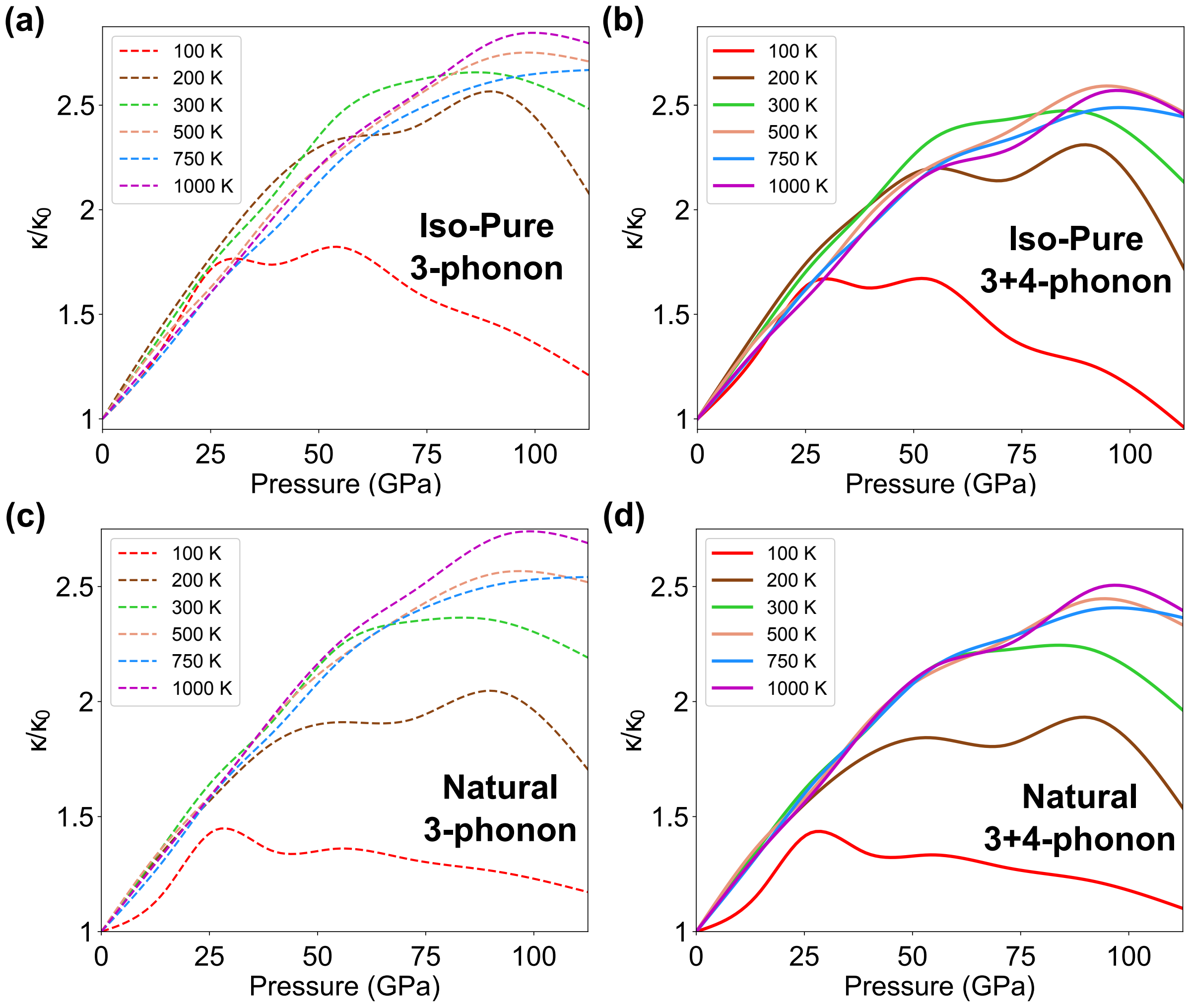

Supplementary Figure 12. **Pressure dependence of the κ of 3C-SiC at different temperatures.** Pressure dependence of the calculated κ of isotopically enriched and naturally occurring cubic 3C-SiC scaled by its zero pressure values for different temperatures. Dashed curves are for three-phonon scattering only; solid curves are for three- and four-phonon scattering.

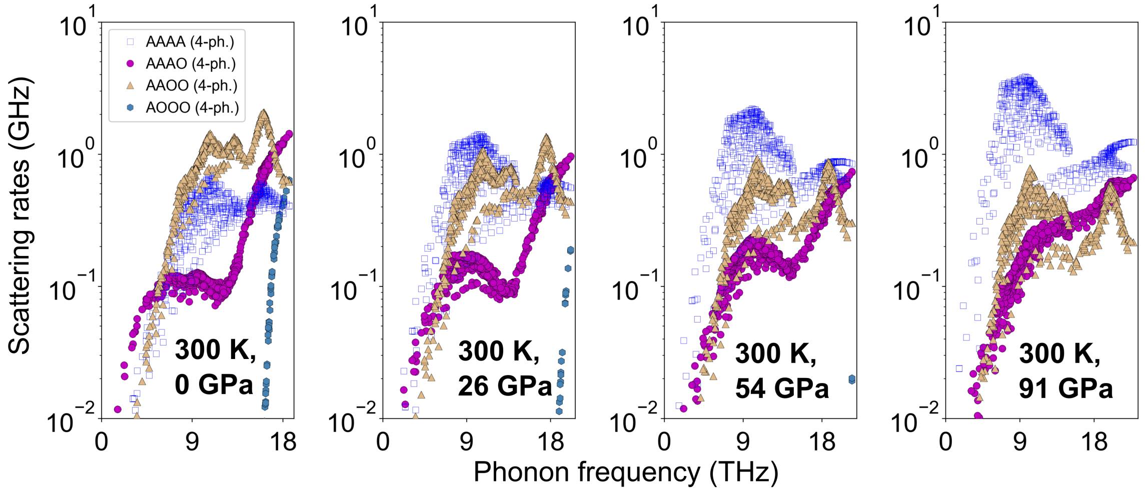

Supplementary Figure 13. **Process-wise four-phonon scattering rates for 3C-SiC.** Process-wise four-phonon scattering rates for 3C-SiC at $T = 300$ K and different pressures. AAAA scattering rates increase with increasing P and dominate at high P, qualitatively similar to the behavior in BP.

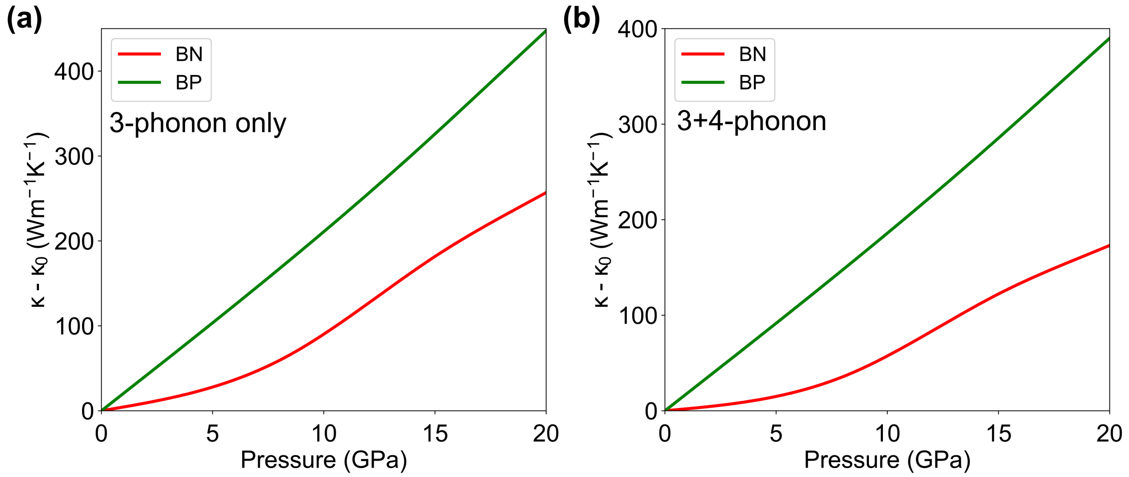

Supplementary Figure 14. **Comparison of the pressure dependence of κ for BN and BP.** Three-phonon **(a)** and 3+4-phonon **(b)** limited κ of BN and BP as functions of pressure at room temperature, relative to their corresponding values under ambient pressure (κ_0). Even though BN is lighter in mass, BP has the largest rate of rise in κ.

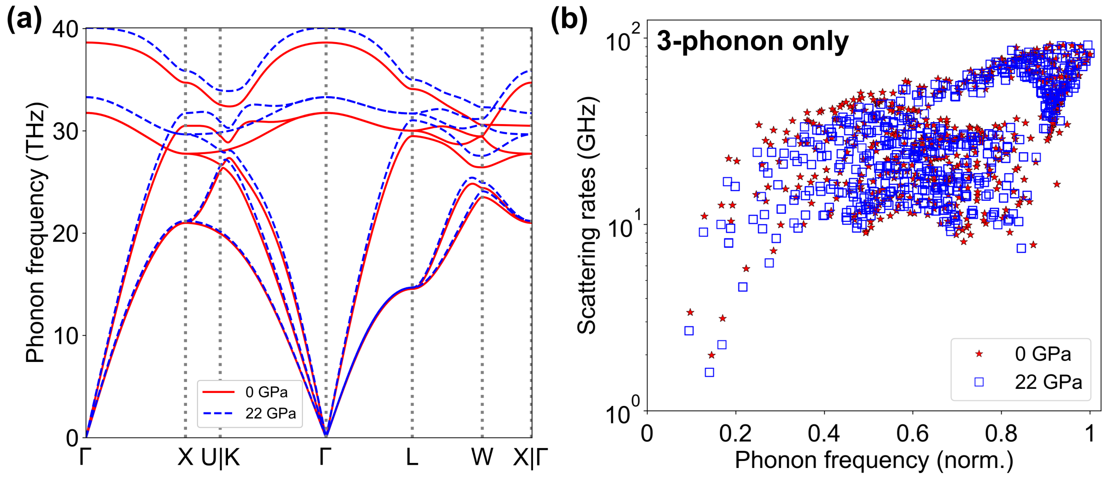

Supplementary Figure 15. **Pressure dependence of phonon dispersions and three-phonon scattering rates of BN. (a)** Phonon dispersions and **(b)** three-phonon scattering rates of BN at 300 K and at two different pressures - 0 GPa and 22 GPa. There is no A-O frequency gap in BN, even at high pressures. The κ of BN increases with increasing pressures (at low pressure) due to the slightly stiffening acoustic phonon frequencies (which results in a slight increase in the acoustic phonon group velocities) and a slightly weakening three-phonon scattering rates with increasing pressure.

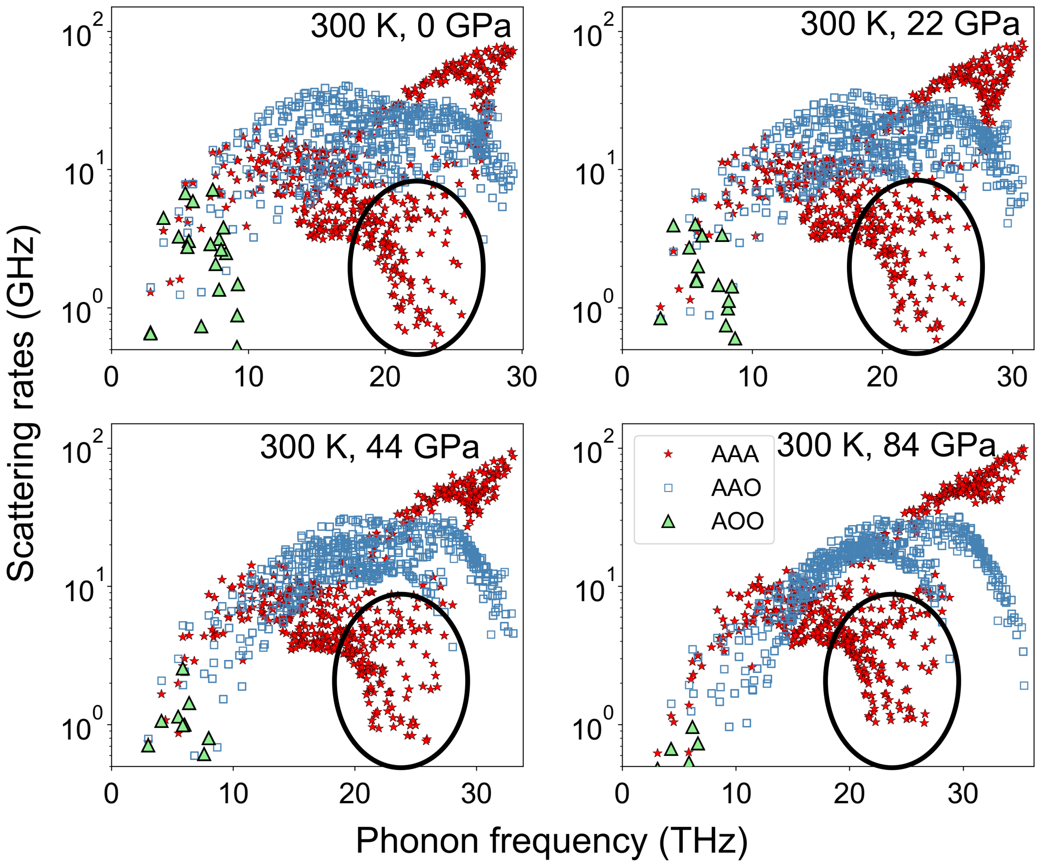

Supplementary Figure 16. **Processwise three-phonon scattering rates of BN at room temperature and different pressures.** AAA, AAO and AOO three-phonon scattering rates for cubic BN as functions of phonon frequencies at different pressures and room temperature. Similar to BP, the AAA scattering rates are weakened by the AAA selection rule due to the bunching-together of the acoustic phonons, and are anomalously weak (highlighted by the black ovals) in BN, as described in the manuscript. However, unlike BP, the AAO scattering rates completely dominate these anomalously weak AAA scattering rates, since there is no frequency gap between the acoustic and the optic phonons (A-O gap) in BN. This results in overall strong three-phonon scattering rates and negligible competition between AAA and AAO scattering channels with increasing pressure.

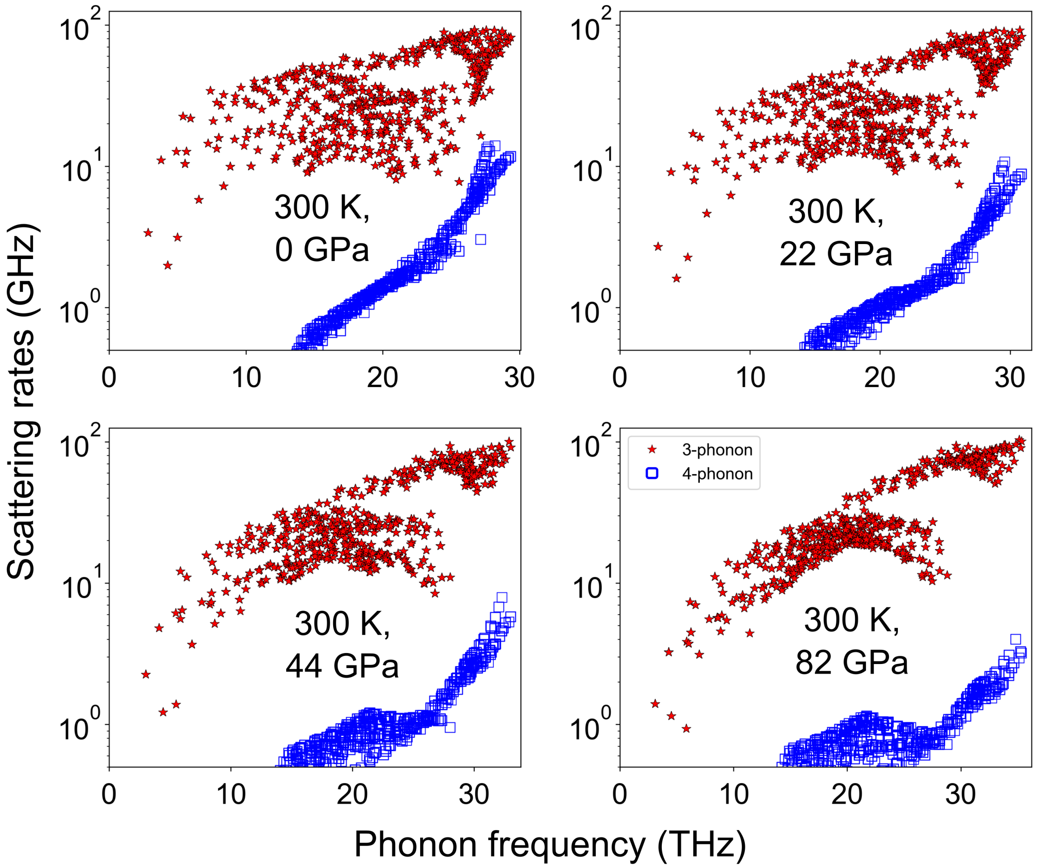

Supplementary Figure 17. **Three-phonon vs. four-phonon scattering rates for cubic BN as functions of phonon frequencies at different pressures and room temperature.** Four-phonon scattering rates are much weaker than the three-phonon scattering rates at room temperature and all pressures considered here.
